# The Elicitation Effect of Pathogenic Fungi on Trichodermin Production by *Trichoderma brevicompactum*

**DOI:** 10.1155/2013/607102

**Published:** 2013-12-05

**Authors:** Xu-Ping Shentu, Wei-Ping Liu, Xiao-Huan Zhan, Xiao-Ping Yu, Chuan-Xi Zhang

**Affiliations:** ^1^Institute of Insect Science, Zhejiang University, Hangzhou, Zhejiang 310058, China; ^2^College of Life Science, China Jiliang University, Zhejiang Provincial Key Laboratory of Biometrology and Inspection & Quarantine, Hangzhou, Zhejiang 310018, China

## Abstract

The effects of six species of phytopathogenic fungi mycelia as elicitors on trichodermin yield by *Trichoderma brevicompactum* were investigated. Neither nonviable nor viable mycelia of *Botrytis cinerea*, *Alternaria solani*, *Colletotrichum lindemuthianum,* and *Thanatephorus cucumeris* demonstrated any elicitation on the accumulation of trichodermin. However, the production of trichodermin was increased by the presence of viable/nonviable *Rhizoctonia solani* and *Fusarium oxysporum* mycelia. The strongest elicitation effect was found at the presence of nonviable *R. solani*. At the presence of nonviable *R. solani*, the maximum yield of trichodermin (144.55 mg/L) was significantly higher than the Control (67.8 mg/L), and the cultivation time to obtain the maximum yield of trichodermin decreased from 72 h to 60 h. No difference of trichodermin accumulation was observed by changing the concentration of nonviable *R. solani* from 0.1 to 1.6 g/L. It was observed that the optimum time for adding nonviable *R. solani* is immediately after inoculation. The diameter of *T. brevicompactum* mycelial globule after 72 h cultivation with nonviable *R. solani* elicitor was smaller than that of the Control.

## 1. Introduction

Trichothecenes are a large group of sesquiterpenoid-derived secondary metabolites and have mainly been isolated from species of* Fusarium* and other fungal genera like *Stachybotrys*, *Myrothecium*,* Trichoderma, *and *Trichothecium* [1, 2]. To date, more than 200 trichothecenes have been reported and are divided into four types (A–D) according to their chemical structures [3–6]. Trichodermin biosynthesized by *Trichoderma *belongs to type A [7]. Trichodermin had stronger antifungal activity against *Saccharomyces cerevisiae*, *Kluyveromyces marxianus*, *Candida albicans*, *C. glabrata*,* C. tropicalis,* and *Aspergillus fumigatus* as compared to amphotericin B and hygromycin [1]. Furthermore, trichodermin had a potential biological control activity on the phytopathogenic fungi such as *Botrytis cinerea* and* Rhizoctonia solani* [8, 9]. It is also a potent inhibitor of protein synthesis in mammalian cells [10].

Recent researches on trichodermin mainly focused on the biosynthetic pathway in *Trichoderma *and its antifungal activities [1, 9–13]. At present, the related report about improvement on trichodermin yield is very scarce and fermentation units were only up to 325 *μ*g/L [14]. To enable large-scale production, it is important to investigate the influence of different factors on yield and optimize the fermentation conditions so as to improve the yield of trichodermin. In general, the methods for enhancing the yield of secondary metabolites biosynthesized by microorganisms are based on strain improvement, the adjustment of bioreactor conditions, and precursor/elicitor input. In recent years, the production of pharmaceutically important secondary metabolites has been effectively increased by the addition of fungal elicitors, such as mycelia, carbohydrate fragments, proteins, oligosaccharides, or secretions derived from fungi in certain plant cell cultures [15–17]. However, the improvement on trichodermin yield in *Trichoderma* using precursor/elicitor has not been reported.

In the present work, the elicitation effects of six viable and nonviable phytopathogenic fungi species on trichodermin production by *T. brevicompactum *were studied. The concentration and time for adding fungal elicitor were optimized. The morphological characteristics of *T. brevicompactum* mycelia with/without elicitors were also compared.

## 2. Materials and Methods

### 2.1. Fungal Strains

Fungal strains, *T. brevicompactum*,* R. solani*, *Fusarium oxysporum*,* B. cinerea*, *Alternaria solani*, *Colletotrichum lindemuthianum, *and* Thanatephorus cucumeris,* were maintained on potato dextrose agar (PDA) slants at 4°C for further tests. The biological agent, *T. brevicompactum,* was isolated from plant garlic (*Allium sativum*). The above phytopathogens were kindly provided by the Institute of Plant Protection and Microbiology, Zhejiang Academy of Agricultural Sciences.

### 2.2. Preparation of *T. brevicompactum* Spores

*T. brevicompactum* was cultured on PDA medium in the 9 cm diameter Petri dishes at 28°C. After 1 week, 5 mL of sterile water was added to the Petri plate culture. The spores were gently dislodged from the surface with a sterile glass rod and the suspension was filtered through filter paper to remove mycelia fragments. Subsequently, the suspension was diluted with sterile water and adjusted to the concentration about 1.0 × 10^6^ conidia/mL by a thrombocytometer [18].

### 2.3. Trichodermin Analysis by Gas Chromatographic (GC)

The trichodermin was quantified by GC (Agilent 6890N) with an HP-5 capillary column (5% phenyl methyl siloxane, 30 m × 320 *μ*m × 0.25 *μ*m), an FID detector, and nitrogen as the carrier gas. The injector and the detector temperatures were 250°C and 270°C, respectively, and the column temperature was raised by program with an initial temperature of 100°C maintaining for 5 min and an increase at a rate of 10°C/min up to 260°C maintaining for 5 min. The flow rate of N_2_ was 1.5 mL/min [19].

### 2.4. Effect of Viable Mycelia on Trichodermin Yield

Spores (1 mL) and one-round plug with 4 mm diameter excised from the growing edge of a 4-day-old phytopathogenic fungal colony were coinoculated into the same 300 mL Erlenmeyer flask containing 60 mL sterilized medium. The medium consisted of 8 g/L dextrose, 10 g/L beef extract, and 2 g/L sodium chloride. After different incubation times on a rotary shaker at 180 rpm under 28 ± 1°C, the fermentation broth was separated from the mycelia by filtration using a Buchner funnel. The filtered broths were extracted exhaustively with ethyl acetate (v/v, 1 : 2). The organic fractions were combined and evaporated to dryness in a vacuum at 50°C. The recovered residues were resuspended in methanol and subjected to GC analysis for the quantification of trichodermin. The culture of *T. brevicompactum* without adding fungal pathogens was served as the Control.

### 2.5. Effect of Nonviable Mycelia on Trichodermin Yield

The mycelia of phytopathogenic fungus were picked from slants and inoculated into 300 mL Erlenmeyer flasks containing 60 mL PD medium. After 5 days of cultivation on a rotary shaker at 180 rpm under 28 ± 1°C, the mycelia were separated by filtration using a Buchner funnel. Then the mycelia were drained and freeze-dried at −54°C and under 0.5 mbar vacuum for 12 h. The dehydrated mycelia were grounded in a mortar to obtain the fine powder. It was proved that the powder obtained by the above procedure was unable to grow when inoculated in minimal or rich medium. The culture of *T. brevicompactum* without mycelia powder was used as the Control.

### 2.6. Effects of the Concentration and Time for Adding Nonviable *R. solani* on Trichodermin Production

The influence of nonviable *R. solani* concentration ranging from 0.1 to 1.6 g/L on trichodermin accumulation was studied. Zero h and 12 h after inoculation were designed to test the effects of the time for adding nonviable *R. solani* on trichodermin yield. After cultivation, the mycelia of *T. brevicompactum* were separated by filtration using a Buchner funnel. Then the mycelia were dried at 90°C until reaching a constant weight. The dry mycelia were weighted using a digital balance (HA1001, Shanghai Electronic Balance Co., Shanghai, China).

### 2.7. Statistical Analysis

The whole statistical analysis was performed using SPSS 15.0 software. Trichodermin concentration comparisons between different treatments were made using one-way analysis of variance (ANOVA) followed by LSD multiple comparison test. *P* values <0.05 were considered as significant.

## 3. Results

### 3.1. Influence of Phytopathogenic Fungi on Trichodermin Production

**Figure 1:**
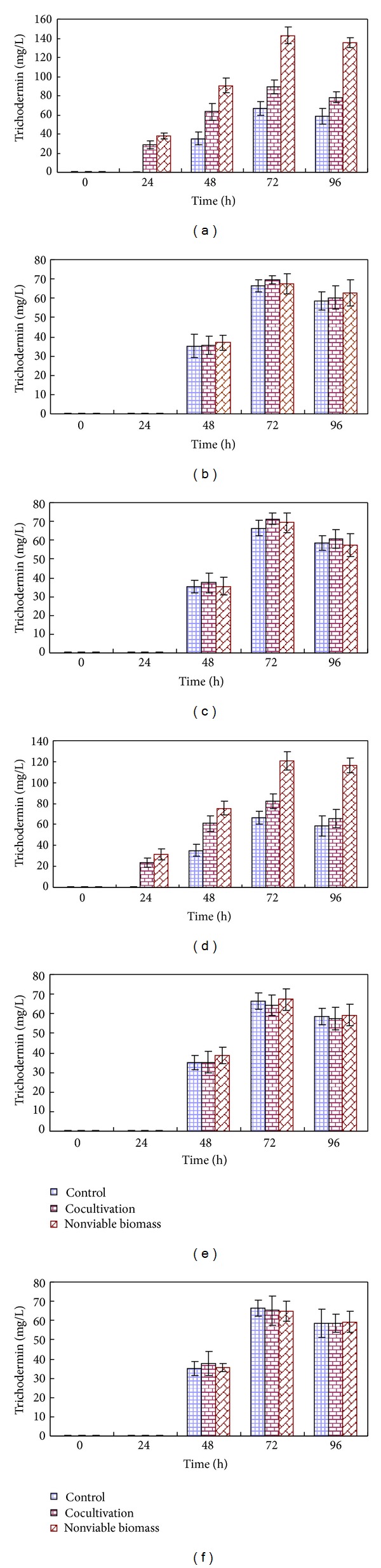
Trichodermin production by *T. brevicompactum* at the presence of pathogenic fungi. (a) *R. solani*, (b) *B. cinerea*, (c) *T. cucumeris*, (d) *F. oxysporum*, (e) *A. solani*, (f) *C. lindemuthianum*.

The effects of six species of phytopathogenic fungi mycelia as elicitors on trichodermin yield were tested. Neither nonviable nor viable mycelia of *B. cinerea*, *C. lindemuthianum*,* T. cucumeris,* or *A. solani* caused significant differences in the accumulation of trichodermin as compared with the Control (Figure 1). The production of trichodermin by *T. brevicompactum* in liquid cultures was differently affected by the presence of viable/nonviable *R. solani* or *F. oxysporum* mycelia. No significant differences of trichodermin yields were observed with viable *R. solani* and *F. oxysporum* as elicitors, but a significantly higher concentration of trichodermin was produced with viable *R. solani* and *F. oxysporum* compared with Controls at 24 h, 48 h, 72 h, and 96 h, respectively. Similarly, a distinctly higher concentration of trichodermin occurred at the presence of nonviable *R. solani* or *F. oxysporum* mycelia as compared to the Control. In particular, the production of trichodermin in liquid cultures at the presence of nonviable *R. solani* was two times higher than the Control at 72 h. However, the elicitation effect of nonviable *F. oxysporum* on trichodermin accumulation was not as obvious as that of *R. solani*.

### 3.2. Influence of the Concentration of Nonviable *R. solani* Mycelia on Trichodermin Production

**Figure 2:**
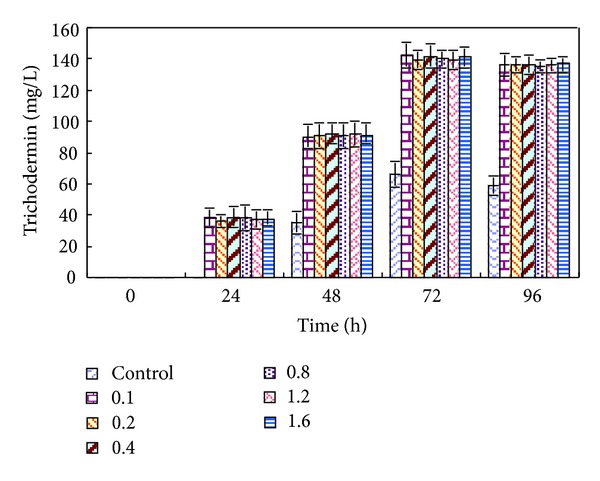
Influence of the concentration for nonviable *R. solani *(g/L) on trichodermin production.

**Figure 3:**
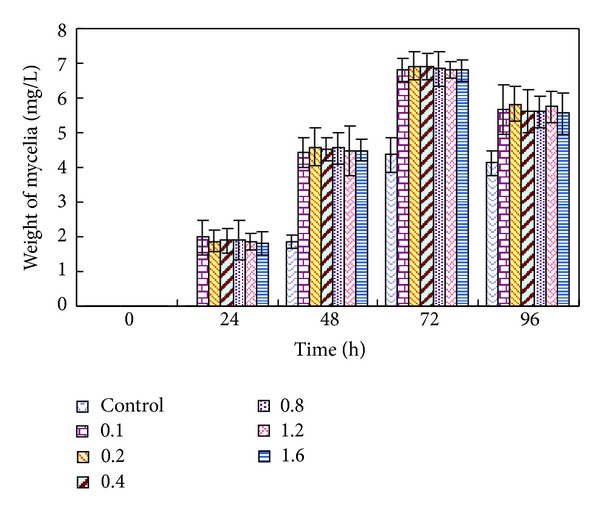
Influence of the concentration for nonviable *R. solani *(g/L) on *T. brevicompactum* growth.

The influence of nonviable *R. solani* mycelia with different concentrations ranging from 0.1 to 1.6 g/L on trichodermin accumulation was studied. The results showed that there was no distinct difference among the yields of trichodermin by adding different concentrations of non-viable *R. solani* mycelia with the same cultivation times (Figure 2). That is to say, within the concentration range investigated, nonviable *R. solani* had a positive effect on trichodermin yield in spite of its concentration. Moreover, more biomass was obtained under the elicitation of *R. solani* elicitor (Figure 3).

### 3.3. Effect of the Time for Adding Nonviable *R. solani* Mycelia on Cultivation Process

**Figure 4:**
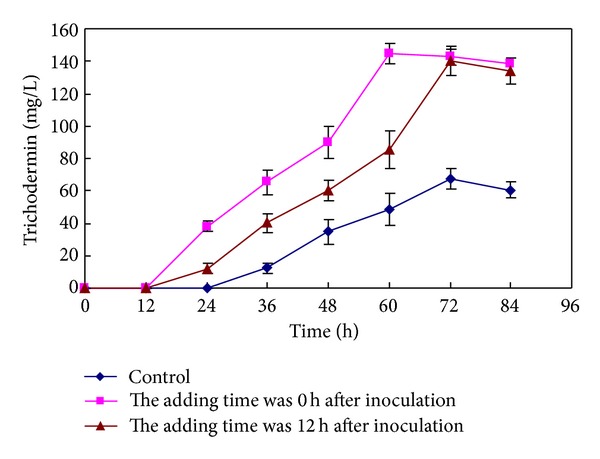
Influence of the time for adding nonviable *R. solani* mycelia (0.1 g/L) on trichodermin biosynthesis.

The nonviable *R. solani* mycelia as an elicitor significantly influenced the production of trichodermin biosynthesized by *T. brevicompactum* as compared with the Control (Figure 4). When the elicitor was added at the same time as inoculation, trichodermin was detected in fermentation broth after 12 h using the established GC method; however, for the Control, trichodermin was not found until 24 h. The results indicate that trichodermin biosynthesis was advanced by *T. brevicompactum*. After adding non-viable* R. solani* mycelia, *T. brevicompactum* yielded a significantly higher trichodermin production (144.55 mg/L) as compared to the Control (67.8 mg/L), and the cultivation time to obtain the maximum yield decreased from 72 h to 60 h. Adding *R. solani* not only improved the yield of trichodermin but also shortened the culture period. When elicitors were added 12 h after inoculation, the final yield of trichodermin was also enhanced but trichodermin biosynthesis was not advanced compared to Control.

### 3.4. Morphological Change of *T. brevicompactum *at the Presence of Nonviable *R. solani*

After 72 h cultivation, the mycelia were obtained from the fermentation broth by filtration using a Buchner funnel. Mycelial globules were observed in the presence of nonviable *R. solani* or the Control. The dry weights of mycelia for the nonviable *R. solani* group and the Control were significantly different. Furthermore, the diameter of mycelial globule for nonviable *R. solani *group was smaller than that of the Control.

## 4. Discussion

It is well known that the main bottleneck of secondary metabolites production is the low production in fermentation [20]. So, many research efforts have been focused on improving the yield of the active secondary metabolites. However, the usage of different elicitors has not been comprehensively explored in trichodermin production.

Encouraging results were obtained that the mycelia of *R. solani* and *F. oxysporum* as elicitors can significantly promote the trichodermin production in our experiments. On the contrary, the mycelia from other tested fungi, *B. cinerea, C. lindemuthianum*,* T. cucumeris* or, *A. solani,* did not show elicitation effect on trichodermin yield. Our research results were similar to previous researches [21], where the yields of antifungal compounds T39 butenolide and harzianopyridone produced by commercial strain *T. harzianum* T39 were improved in cocultures with *B. cinerea* or *R. solani*. Similarly, nonviable *R. solani* was found to be able to increase the accumulation of 6-pentyl-a-pyrone (6PP) by *T. harzianum* [22]. In this work, it was demonstrated that adding mycelia of pathogenic fungi into the cultivation of *Trichoderma* could improve the production of active secondary metabolites. The genus *Trichoderma* is widely used as a biocontrol agent for inhibiting fungal phytopathogens growth by three mechanisms: competition, parasitism, and antibiosis [23, 24]. Previous studies suggested that mycoparasitic behaviour is induced by the responses triggered by molecules released from the host or located on its surface [25, 26]. Furthermore, the studies underlying signal transduction pathways found that a loss of mycoparasitic abilities in *Trichoderma* mutant was reflected strongly by the decreased chitinase activities and reduced production of active metabolites (e.g., 6pp) [26–28]. These research results implied that mycoparasitic behaviour of *Trichoderma* against phytopathogenic fungi, induced by molecules from their hosts, can promote active compounds production by *Trichoderma*. Therefore, the results in this work suggest that induced molecules from *R. solani*/*F. oxysporum* can induce mycoparasitic behaviour and further stimulate trichodermin biosynthesis by *T. brevicompactum*. Certainly, the correlation among induced molecules, mycoparasitism, and metabolites biosynthesis by *Trichoderma* needs to be further studied in order to clarify the elicitation mechanism.

However, nonviable *R. solani *and *F. oxysporum* mycelia as elicitors had a strong effect on trichodermin production than viable *R. solani *and *F. oxysporum* mycelia. Our other researches (unpublished data) indicated that *R. solani* and *F. oxysporum* could inhibit *T. brevicompactum* growth. So, it is understandable that the inhibition on *T. brevicompactum* by viable *R. solani* or* F. oxysporum* decreased the elicitation effect in cultivation.

In our study, mycelia morphology of *T. brevicompactum* was also investigated. The mycelial globule of *T. brevicompactum* with elicitor was smaller in diameter than that of the Control. The potential advantage of a smaller mycelial globule is that it can facilitate nutrition absorption and metabolites secretion during cultivation.

The results indicate that nonviable *R. solani* is an effective and convenient elicitor in trichodermin production by *T. brevicompactum*. The direct application of phytopathogenic fungi as elicitors may represent a novel approach to improve bioactive compounds yield by *Trichoderma*. Certainly, more researches such as the structure of induced molecules in *R. solani* and the molecular evidence of elicitation are necessary to better understand and use this novel approach in antifungal compounds production.

## References

[1] Tijerino A, Cardoza RE, Moraga J (2011). Overexpression of the trichodiene synthase gene tri5 increases trichodermin production and antimicrobial activity in *Trichoderma brevicompactum*. *Fungal Genetics and Biology*.

[2] Tate WP, Caskey CT (1973). Peptidyltransferase inhibition by trichodermin. *The Journal of Biological Chemistry*.

[3] Grove JF (2007). The trichothecenes and their biosynthesis. *Fortschritte der Chemie organischer Naturstoffe*.

[4] Kimura M, Tokai T, Takahashi-Ando N, Ohsato S, Fujimura M (2007). Molecular and genetic studies of *Fusarium* trichothecene biosynthesis: pathways, genes, and evolution. *Bioscience, Biotechnology and Biochemistry*.

[5] Ueno Y (1984). Toxicological features of T-2 toxin and related trichothecenes. *Fundamental and Applied Toxicology*.

[6] Ueno Y (1985). The toxicology of mycotoxins. *Critical Reviews in Toxicology*.

[7] Kraus GF, Druzhinina I, Gams W (2004). *Trichoderma brevicompactum* sp. nov. *Mycologia*.

[8] Shentu XP, Shi YJ, Yu XP (2010). Identification of an endophytic isolate from *Ilex cornuta* and its antagonism against *Rhizoctonia solani*. *Chinese Journal of Pesticide Science*.

[9] Yin GL, Wang WM, Sha S, Liu L, Yu XP (2010). Inhibition and control effects of the ethyl acetate extract of *Trichoderma harzianum* fermented broth against *Botrytis cinerea*. *African Journal of Microbiology Research*.

[10] Carrasco L, Barbacid M, Vazquez D (1973). The trichodermin group of antibiotics, inhibitors of peptide bond formation by eukaryotic ribosomes. *Biochimica et Biophysica Acta*.

[11] Cardoza RE, Malmierca MG, Hermosa MR (2011). Identification of loci and functional characterization of trichothecene biosynthesis genes in filamentous fungi of the genus *Trichoderma*. *Applied and Environmental Microbiology*.

[12] Tijerino A, Hermosa R, Cardoza RE (2011). Overexpression of the *Trichoderma brevicompactum tri5* gene: effect on the expression of the trichodermin biosynthetic genes and on tomato seedlings. *Toxins*.

[13] Degenkolb T, Dieckmann R, Nielsen KF (2008). The *Trichoderma brevicompactum* clade: a separate lineage with new species, new peptaibiotics, and mycotoxins. *Mycological Progress*.

[14] Bertagnolli BL, Daly S, Sinclair JB (1998). Antimycotic compounds from the plant pathogen *Rhizoctonia solani* and its antagonist *Trichoderma harzianum*. *Journal of Phytopathology*.

[15] Wang GP, Zheng BQ, Zhou ZZ, Zhang CL (2010). Optimization of fermentation conditions for trichodermin by the mutant strain UL 60-1 *Trichoderma. Taxi*. *Chinese Journal of Biological Control*.

[16] Zheng W, Zhao Y, Zheng X (2011). Production of antioxidant and antitumor metabolites by submerged cultures of *Inonotus obliquus* cocultured with *Phellinus punctatus*. *Applied Microbiology and Biotechnology*.

[17] Liang CX, Li YB, Xu JW (2010). Enhanced biosynthetic gene expressions and production of ganoderic acids in static liquid culture of Ganoderma lucidum under phenobarbital induction. *Applied Microbiology and Biotechnology*.

[18] Rabea EI, Badawy MEI, Steurbaut W, Stevens CV (2009). In vitro assessment of N-(benzyl)chitosan derivatives against some plant pathogenic bacteria and fungi. *European Polymer Journal*.

[19] Shentu XP, Shi YJ, Yu XP (2008). Identification and quantification of trichodermin in fermentation by GC. *Chinese Pharmaceutical Journal*.

[20] Berger RG (1995). *Aroma Biotechnology*.

[21] Vinale F, Ghisalberti EL, Sivasithamparam K (2009). Factors affecting the production of *Trichoderma harzianum* secondary metabolites during the interaction with different plant pathogens. *Letters in Applied Microbiology*.

[22] Serrano-Carreón L, Flores C, Rodríguez B, Galindo E (2004). *Rhizoctonia solani*, an elicitor of 6-pentyl-*α*-pyrone production by *Trichoderma harzianum* in a two liquid phases, extractive fermentation system. *Biotechnology Letters*.

[23] Harman GE (2006). Overview of mechanisms and uses of *Trichoderma* spp. *Phytopathology*.

[24] Abdel-Fattah GM, Shabana YM, Ismail AE, Rashad YM (2007). *Trichoderma harzianum*: a biocontrol agent against *Bipolaris oryzae*. *Mycopathologia*.

[25] Omero C, Inbar J, Rocha-Ramirez V, Herrera-Estrella A, Chet I, Horwitz BA (1999). G protein activators and cAMP promote mycoparasitic behaviour in *Trichoderma harzianum*. *Mycological Research*.

[26] Reithner B, Brunner K, Schuhmacher R (2005). The G protein *α* subunit Tga1 of *Trichoderma atroviride* is involved in chitinase formation and differential production of antifungal metabolites. *Fungal Genetics and Biology*.

[27] Sivasithamparam K, Ghisalberti EL (1998). *Trichoderma and Gliocladium*.

[28] Wiest A, Grzegorski D, Xu BW (2002). Identification of peptaibols from *Trichoderma virens* and cloning of a peptaibol synthetase. *The Journal of Biological Chemistry*.

